# Reduction of Acute Postoperative Pain With Pre-Emptive Pregabalin Following Laparoscopic Cholecystectomy

**DOI:** 10.7759/cureus.27783

**Published:** 2022-08-08

**Authors:** Fahad Najam, Nusrat Jafri, Mohammad Nazim Khan, Umar Daraz

**Affiliations:** 1 Anaesthesiology, Abbasi Shaheed Hospital, Karachi, PAK; 2 Pain Management, Abbasi Shaheed Hospital, Karachi, PAK; 3 Critical Care, Abbasi Shaheed Hospital, Karachi, PAK

**Keywords:** acute care surgery and trauma, pain man, preemptive, acute pain, pain

## Abstract

Abstract

Pregabalin has been considered to be a safe treatment for neuropathic pain. Owing to the lack of research regarding the use of pregabalin in the management of pain in under-resourced settings, our study aimed to deduce the effectiveness of a pre-emptive single dose of pregabalin pre-operatively to provide pain relief after laparoscopic cholecystectomy. Treating acute pain is essential to avoid an increased hospital stay. There is a need for non-opioid drugs with lower risks to avoid using opioids, which lead to many side effects.

Methodology

Patients diagnosed with cholelithiasis and scheduled to undergo laparoscopic cholecystectomy at the Abbasi Shaheed Hospital were included in this study. The study aimed to determine whether the effect of pregabalin in combination with patient-controlled analgesia can decrease pain scores. This was a double-blind study where patients, caregivers, and analysts were blinded to group allocation and drugs administered until the data was recorded and sealed. The patients were divided into pregabalin and placebo groups through a web-based model; blocks of four were used and stratification was employed at the center. A confidence interval of 95% was considered significant.

Results

In our study, a total number of 60 patients were included. They were randomly divided by a computer-based model into two groups, the pregabalin group, and the control group. The placebo group had 33 patients while the pregabalin group had 27 patients. The pregabalin group was given a pregabalin tablet of 150 mg before surgery while the placebo group was given an identical-looking placebo. Patient-controlled analgesia was started in both groups and the visual analog scale (VAS) scoring was observed postoperatively. The pregabalin group had a decreased incidence of pain as compared to the placebo group. There were no significant side effects during the trial; episodes of vomiting were managed using intravenous ondansetron.

Conclusion

Pregabalin is effective in reducing pain in an acute postoperative period when compared with a placebo. Patients who were pre-emptively administered pregabalin reported decreased VAS as compared to the placebo. However, both were inefficient in reducing postoperative nausea and vomiting.

## Introduction

In settings with few resources, managing acute post-operative pain is very difficult owing to the low availability of medical staff and drugs. Acute post-operative pain is the most serious complaint which requires the use of opioids and non-opioid therapies. Opioid therapy in the acute postoperative period can have many side effects such as nausea, vomiting, pruritus, and respiratory depression [1-3]. Many clinicians now consider the use of non-opioid therapy in combination with a multimodal regimen for postoperative pain management. This regimen is gaining popularity in under-resourced settings because of its decreased side effects [4].

Pregabalin is part of the gabapentinoid group, which decreases the release of excitatory neurotransmitters substance P, serotonin, and glutamate, leading to a decrease in post-operative pain. Pregabalin is known to reduce post-operative pain and prevent hyperalgesia. Previous studies have shown that pregabalin can reduce pain in the acute post-operative period and that its pre-emptive use decreased overall fentanyl consumption and reduced post-operative pain [5].

The goal of our study was to analyze the effect of pregabalin in combination with patient-controlled analgesia to provide adequate pain relief in the acute post-operative period. A dose of 150 mg was used since it was effective and had minimum side effects. We aimed to determine its role in reducing VAS scores and incidence of post-operative nausea and vomiting. Both factors can adversely affect the recovery of a patient and prolong hospital stay [6].

## Materials and methods

After approval of the ethical review board (IRB #23545), permission was taken to conduct a double-blind randomized trial at Abbasi Shaheed Hospital. All patients selected were adults aged between 25 and 40. The patients were admitted a day before surgery. The consent of all the participants was taken and the procedure was explained to them.

Patients were divided randomly by a computer-based model into two groups, the pregabalin group, and the control group. A total of 60 patients were included in our study to keep the confidence interval at 95%, the analysis failure below 20%, the alpha value of 0.05, and the beta value of 0.08. The study was double-blinded in which the patients, caregivers, and those recording the data were blinded to the treatment. Only after completion of the data record, the treatment was revealed. The patients were divided into the pregabalin group and placebo group through a web-based model where blocks of four were used and stratification was employed at the center. Patients included in the study were ASA 1 and 2; they had no uncontrolled systemic disease.

The primary objective of our study was to determine whether pregabalin before surgery could provide adequate pain relief in combination with patient-controlled analgesia using fentanyl. Our second objective was to analyze the effectiveness of pregabalin in reducing the incidence of post-operative nausea and vomiting in patients. The study team kept all the logs of all the patients who had met the eligibility criteria and those who left the trial (on request for early discharge); the reason for discharge was also noted

The pregabalin group patients had received 150 mg of pregabalin six hours before surgery. The control group had been given a placebo six hours before surgery.

The sample size was calculated by taking the alpha value of 0.05 and the beta value of 0.08. Twenty-five people were enrolled in each group for the result to be significant. Inclusion criteria required the patients to have no uncontrolled systemic diseases such as hypertension and diabetes. They had no previous abdominal surgery, and all were of ASA I and ASA II. Exclusion criteria were the presence of uncontrolled systemic diseases, psychiatric illness, or chronic pain.

All patients were given a similar anesthesia technique. Induction was done with propofol 150 mg and atracurium 40 mg. Endotracheal intubation was done after laryngoscopy, and the endotracheal tube was properly secured. Anesthesia was maintained with halothane at 1.0 Mac. Additionally, nalbuphine 10 mg was given intravenously as part of a multimodal regimen. The reversal was done using a combination of glycopyrrolate 0.001 mg/kg and neostigmine 0.05 mg/kg to antagonize the neuromuscular block.

All patients were transferred to the post-anesthesia care unit, where pain scores and intensity of post-operative nausea and vomiting were monitored by an independent anesthetist who was blinded to group distribution.

Fentanyl patient-controlled analgesia was started at 25 micrograms with a lockout time of 10 minutes. The maximum dose allowed was 1.9 micrograms per kg per pain measure at intervals of 0 to 3 hours, 3 to 6 hours, and 6 to 12 hours. Post-operative nausea and vomiting were monitored with the criteria of no nausea, mild, moderate, and severe. All patients with mild nausea and vomiting were given ondansetron intravenous 4 mg as rescue therapy.

Data were analyzed using SPSS version 26 (IBM Corp., Armonk, NY). After normality assessment for quantitative variables, the mean with standard deviation was reported for the age and weight of the participants. For the duration of the surgery, heart rate and baseline mean arterial pressure (MAP) median with interquartile ranges were calculated. The VAS pain scale along with post-operative nausea and vomiting scores were categorized and reported with frequencies and percentages. Both groups, i.e., the placebo and pregabalin groups, were compared. Age and weight were compared using an independent t-test while the Mann-Whitney U test was applied for other quantitative variables. A chi-square test was used after assessing the assumptions for all the qualitative variables except post-operative nausea and vomiting at six hours, for which Fisher’s exact test was used. A p-value of less than 0.05 was considered significant.

## Results

**Figure 1:**
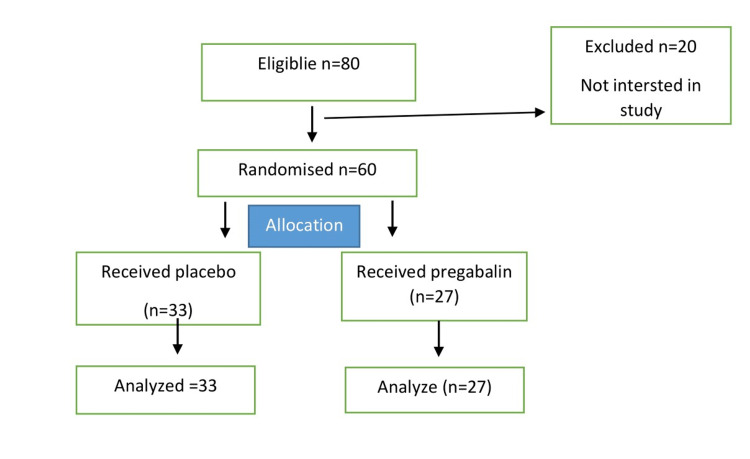
Consolidated standards of reporting trials (CONSORT) flow chart

Consolidated standards of reporting trials (CONSORT) flow chart showing stages of allocation between the two groups is shown in Figure 1.

A total of 60 participants were enrolled in the study, of which 33 were part of the placebo group while 27 were in the intervention group and were given pregabalin pre-surgery. As shown in Table 1, the mean age and weight along with the median duration of surgery, baseline MAP, and heart rate at 3, 6, and 12 hours were similar in both groups, while only the baseline heart rate was found to be slightly raised in the placebo group but was in the normal range (p=0.028).

**Table 1:** Parameters observed between the placebo and pregabalin groups MAP: mean arterial pressure

		Group A Placebo	Broup B Pre-Gabalin	Total	p-Value	Test Applied
Age (years)	Mean(SD)	44.3(6.1)	45.0(6.6)	44.6(6.3)	0.457	T-test
Weight (kg)	Mean(SD)	69.0(8.4)	69.5(8.3)	69.2(8.7)	0.596	T-test
Duration_surgery (mins)	Median(IQR)	55(55-58.7)	55(55-56)	55(55-56)	0.661	Mann W -U
Baseline heart rate	Median(IQR)	72(69.3-75)	70(69-70)	70(69-72)	0.028	Mann W -U
Base MAP	Median(IQR)	101(100-103)	100.5(99-102.3)	101(100-103)	0.469	Mann W -U
Heart rate at 3 hr	Median(IQR)	75(74-78)	75(74-75.3)	75(74-78)	0.422	Mann W -U
Heart rate at 6 hr	Median(IQR)	73(69-74.8)	72.5(72-74)	73(71.8-74)	0.827	Mann W -U
Heart rate at 12 hr	Median(IQR)	73(72-74.8)	73.5(70.8-75)	73(72-75)	0.798	Mann W -U
Heart rate at 12 hr	Median(IQR)	73(72-74.8)	73.5(70.8-75)	73(72-75)	1.798	Mann W -U

The comparison between postoperative nausea and vomiting was found to be statistically insignificant in both groups at all times, i.e. at 3 hours, 6 hours, and 12 hours with a p-value of 0.184, 0.72, and 0.697 respectively. However, a highly significant difference was observed among the groups for pain scoring, with a p-value of <0.0001 at all times (Table 2). Interestingly, at all times, the most number of individuals had mild pain in the intervention group, while most individuals in the placebo group had moderate pain at all times. There were no unintended side effects in both groups. Postoperative nausea and vomiting were treated with ondansetron.

**Table 2:** Visual analog score (VAS) and post-operative nausea and vomiting observed postoperatively PONV: postoperative nausea and vomiting

			Group A Placebo	Group B Pre-Gabalin	Total	p-Value
VAS at 3 hours	Mild Pain	N	0	25	25	
		%	0.00%	92.60%	41.70%	<0.0001
	Moderate Pain	N	33	2	35	
		%	100.00%	7.40%	58.30%	
VAS at 6 hours	Mild Pain	N	6	23	29	
		%	18.20%	85.20%	48.30%	<0.0001
	Moderate Pain	N	27	4	31	
		%	81.80%	14.80%	51.70%	
VAS at 12 hours	Mild Pain	N	21	27	48	
		%	63.60%	100.00%	80.00%	<0.0001
	Moderate Pain	N	12	0	12	
		%	36.40%	0.00%	20.00%	
PONV 3 hours	No PONV	N	19	16	35	
		%	57.60%	59.30%	58.30%	0.184
	Mild PONV	N	5	8	13	
		%	15.20%	29.60%	21.70%	
	Moderate PONV	N	9	3	12	
		%	27.30%	11.10%	20.00%	
PONV 6 hours	No PONV	N	9	7	16	
		%	27.30%	25.90%	26.70%	0.72
	Mild PONV	N	16	14	30	
		%	48.50%	51.90%	50.00%	
	Moderate PONV	N	6	6	12	
		%	18.20%	22.20%	20.00%	
			2	0	2	
			6.10%	0.00%	3.30%	
PONV at 12 hours	No PONV	N	27	21	48	
		%	81.80%	77.80%	80.00%	0.697
	Mild PONV	N	6	6	12	
		%	18.20%	22.20%	20.00%	

## Discussion

Pregabalin has been used in previous studies preoperatively to decrease acute postoperative pain after surgery [7]. It has demonstrated an analgesic effect and anti-hyperalgesic effect. After laparoscopic cholecystectomy, post-operative pain can occur as a result of injury to tissues. This then leads to inflammatory mediators causing pan owing to sensitization of central and peripheral receptors. Table 1 shows that the duration of surgery was similar in both groups at around 55 minutes. All patients underwent a similar technique of laparoscopic surgery [8-9].

The use of pregabalin in combination with other non-opioid regimens as part of multimodal analgesia can provide significant pain relief and decrease postoperative pain scores. This leads to a decrease in the duration of hospital stay and reduces complications such as thromboembolism caused by immobility [10-12].

Previous studies have identified that pre-emptive use of pregabalin can reduce overall opioid consumption [13]. This opioid-sparing effect can be useful to avoid complications associated with the use of opioids which include pruritis, nausea, and respiratory depression. In our study, the VAS score at three hours postoperatively shows that the pregabalin group had a lower incidence of mild pain; whereas, most patients in the placebo group reported moderate pain (p<0.001). At the 6th and 12th hours, the postoperative incidence of moderate pain was significantly higher in the placebo group as compared to the pregabalin group. This showed the effectiveness of pregabalin in reducing acute postoperative pain, as seen in Table 2.

Gabapentinoids such as pregabalin can reduce the incidence of postoperative nausea and vomiting [14]. This can be beneficial in hospitals with low resources. With its ability to decrease acute post-operative pain after surgery and decrease the incidence of nausea and opioid-sparing effects, pregabalin should be part of a multimodal regimen in under-resourced settings. In our study, the placebo group had a higher incidence of post-operative nausea and vomiting at the 3rd hour postoperatively compared with the pregabalin group, which reported a lesser episode of nausea and vomiting (p<0.001). However, in the 6th and 12th hours, both groups displayed a similar incidence of post-operative nausea and vomiting, as seen in Table 2.

Many clinicians are of the view that using multimodal techniques enhances recovery and reduces overall hospital stay. Therefore, management of pain becomes vital, and this can be achieved through a multimodal regimen as it employs a different mechanism to reduce actions to reduce post-operative pain. As the sample size of our study was small, large multi-centered and double-blinded studies of longer duration are needed to assess the efficacy of pregabalin in reducing pain and incidence of nausea and vomiting [15-16].

A combination of NSAIDs and pregabalin has been shown to reduce pain scores. Our study has highlighted the beneficial effect of pregabalin on acute post-operative pain after laparoscopic cholecystectomy. The clinical efficacy of pregabalin should be used in under-resourced settings. There were several limitations due to the low resource setting; further randomized control trials are needed to evaluate the effect of long-term pregabalin use in the prevention of hyperalgesia and chronic pain [17-20].

## Conclusions

Our study has demonstrated that pregabalin can provide effective pain relief after acute laparoscopic cholecystectomy. Its clinical efficacy can be used to provide pain relief as a part of a non-opioid pain regimen. Pregabalin can be used as part of multimodal pain management. It is effective in reducing pain in the acute postoperative period, which is demonstrated through lower VAS scores in the pregabalin group compared with the placebo group.
